# George P. Prigatano’s contributions to neuropsychological rehabilitation and clinical neuropsychology: A 50-year perspective

**DOI:** 10.3389/fpsyg.2022.963287

**Published:** 2022-09-09

**Authors:** Alberto García-Molina, George P. Prigatano

**Affiliations:** ^1^Institut Guttmann, Institut Universitari de Neurorehabilitació, Universitat Autònoma de Barcelona, Barcelona, Spain; ^2^Fundació Institut d’Investigació en Ciències de la Salut Germans Trias i Pujol, Barcelona, Spain; ^3^Facultad de Psicología, Centro de Estudios en Neurociencia Humana y Neuropsicología, Universidad Diego Portales, Santiago de Chile, Chile; ^4^Department of Clinical Neuropsychology, Barrow Neurological Institute, Phoenix, AZ, United States

**Keywords:** holistic neuropsychological rehabilitation, psychotherapy, impaired awareness, finger tapping test, clinical neuropsychology

## Abstract

In the 1970s and 1980s, a multitude of cognitive rehabilitation programs proliferated to facilitate recovery after brain injury. However only a few programs provided a framework for ameliorating disturbances in the cognitive, psychological, and interpersonal spheres of the brain-injured patient. Greatly influenced by Leonard Diller and Yehuda Ben-Yishay’s ideas and methods, George P. Prigatano began, in early 1980, a holistic neuropsychological rehabilitation program at the Presbyterian Hospital in Oklahoma City (Oklahoma). The objective of this paper is to summarize the contributions of George P. Prigatano to neuropsychological rehabilitation and clinical neuropsychology during his 50th year of practice. The main body of the paper is structured in three sections. The first section briefly explains the history of neuropsychological rehabilitation in the twentieth century and the emergence of holistic neuropsychological rehabilitation programs in the 1970s. The second section describes the contributions of George P. Prigatano to neuropsychological rehabilitation and clinical neuropsychology (written by AGM). In the third section, the second author (GPP) prepared an autobiographical statement, which attempts to summarize some of the personal and professional experiences which influenced his work. George P. Prigatano’s contributions to neuropsychological rehabilitation and clinical neuropsychology are essential to understanding the therapeutic approaches currently used in the treatment of brain-injured patients.

## Introductory comments

Neuropsychological rehabilitation has a long history as recently documented by García-Molina and Peña-Casanova (2022). The recent passing of two contemporary leaders in this field (i.e., Leonard Diller and Yehuda Ben-Yishay) prompted the first author (AGM) to invite the second author (GPP) to participate in writing a paper documenting his contributions, to date, in the fields of neuropsychological rehabilitation and clinical neuropsychology. The first author (AGM) described, from his perspective, the contributions of George P. Prigatano to these two areas of clinical and academic work. The second author (GPP) prepared an autobiographical statement which attempted to summarize some of the personal and professional experiences which influenced his work.

Typically, descriptions of a person’s contributions to a given field of study are written after their death with no input from them concerning what actually influenced their work. It was the absence of this perspective, in other historical accounts, that prompted the writing of this paper.

## Neuropsychological rehabilitation and the development of holistic approaches

Documents describing techniques and procedures for improving the cognitive functioning of people with brain injuries date back to the seventeenth century (García-Molina and Peña-Casanova, 2022). It was not until the twentieth century, however, that neuropsychological rehabilitation began to develop. The First World War (1914–1918) precipitated the emergence of large-scale efforts at neuropsychological rehabilitation. Persons with traumatic brain injuries (TBIs), typically secondary to gunshot wounds, survived at unprecedented rates given advancements in neurosurgery and the treatment of infections. Governments invested enormous amounts of resources to rehabilitate these surviving soldiers. In countries such as the United States and Germany, it became a national priority.

In 1918 Walter Poppelreuter (1886–1939) published what is considered the first treatise on neuropsychological rehabilitation: *Die Psychischen Schädigungen Durch Kopfschuss im Kriege 1914/17* (Poser et al., 1996). At the same time, Kurt Goldstein (1878–1965) developed a holistic model of neuropsychological rehabilitation (Goldstein, 1939, 1942). He argued that exclusively focusing on cognitive rehabilitation is not adequate. He emphasized the importance of also addressing the personal and social problems faced by war veterans. The aim of neuropsychological rehabilitation, according to Goldstein, was not only to help brain-injured soldiers recover or compensate for their higher integrative brain dysfunctions, but also to help them personally cope with their permanent deficits in a productive manner which reduced the frequency of catastrophic reactions.

The Second World War (1939–1945) provided a renewed impetus for developments in neuropsychological rehabilitation. Oliver Louis Zangwill (1913–1987) addressed the rehabilitation needs of soldiers in England, while Alexander Romanovich Luria (1902–1977) did the same in Russia. Luria and his colleagues suggested that the restoration higher cortical functions could be partially achieved by “de-inhibition” of a temporary inactivated functional system *via* the process of diaschisis (Luria et al., 1969). The processes for achieving this important goal, however, were not easily defined. Luria’s work highlighted the point that understanding the mechanisms of recovery after brain injury not only serves the practical purposes of rehabilitation but also raises important theoretical issues for neuropsychology. Shepherd Ivory Franz (1874–1933), in the United States, made the same point several years earlier.

After the Second World War, the Veterans Administration Hospitals in the United States provided extensive rehabilitation for disabled veterans (integrating speech-language therapy, occupational therapy, physical therapy, psychology, and vocational rehabilitation). Yet holistic programs for neuropsychological rehabilitation had not come into existence.

## Holistic approaches to neuropsychological rehabilitation from the 1970s to 2020s

Between 1974 and 1976, Yehuda Ben-Yishay (1933–2021) and Leonard Diller (1924–2019), at the Rush Institute of Rehabilitation Medicine at New York University School of Medicine, in conjunction with the Israeli government, developed a pilot rehabilitation program for brain-injured Israeli war veterans. Many of Goldstein’s ideas, coupled with the rehabilitation technology Diller had developed with stroke patients and Ben-Yishay’s experience living in Israeli community settings, were successfully applied to these patients. Their demonstration of successful outcomes resulted in a funded 5-year clinical research study in the United States and the establishment of the Rusk Institute Brain Injury Day Treatment Program (New York). Based on the successful outcome of this research study, the program became an established clinical service at the Rusk Institute, one which continues to this day (Ben-Yishay, 1996). The central tenant of this holistic approach is that “to restore a brain-injured individual to function optimally, it is necessary to establish a therapeutic milieu that is experienced by the injured person as “safe” (in the sense that, the chances for the occurrence of “catastrophic” responses by the patient to perceived threats will be reduced). In this “safe” environment, the injured individual can gradually begin to use still intact capacities and learn compensatory skills. The compensation process helps the person attain an enhanced functional adjustment. Improvements in their functional adjustment will facilitate those individuals’ calm acceptance of the life-altering changes, or limitations, that were caused by their brain injury. Acceptance -it is hoped- will also help these individuals to find new meaning in their lives after rehabilitation” (Ben-Yishay and Diller, 2011; p. xii).

Ben-Yishay and Diller’s holistic neuropsychological rehabilitation program provided a framework for ameliorating disturbances in the cognitive, psychological, and interpersonal spheres of the brain-injured patient. The program was organized in a hierarchy of six stages that must be met successfully while undergoing rehabilitation (Ben-Yishay et al., 1985):

• Engagement: The goal is to have the trainee/patient become optimally activated to engage in a purposeful and deliberate manner various remedial tasks.
• Awareness: The goal is to have the trainee/patient become aware of one’s problems, without being overwhelmed by this knowledge.
• Mastery: The goal is to have the trainee/patient learn to compensate for their cognitive deficits.
• Control: The goal is to have the trainee/patient learn to concentrate on the idea behind the act and on its ultimate objective, rather than on the “mechanics,” or how the act is to be carried out.
• Acceptance: The goal is to have the trainee/patient begin to feel that life, as is, is worth living and that they can still derive some pleasure in his/her present life.
• Identity: The goal is to have the trainee/patient learn to reconstitute, to some extent at least, his/her identity.

Ben-Yishay and Diller’s holistic program has been the model for several holistic neuropsychological rehabilitation programs over the world. Between 1979 and 1985, for instance, George P. Prigatano adapted that model to develop a holistic neuropsychological rehabilitation program at the Presbyterian Hospital in Oklahoma City (Oklahoma) (Prigatano et al., 1986). In 1985, he moved to the Barrow Neurological Institute (BNI) (Phoenix, Arizona) to continue this work and establish a Department of Clinical Neuropsychology. Anne-Lise Christensen (1927–2018), influenced by the work of Ben-Yishay and Prigatano, founded a holistic program in 1985, the Center for Hjerneskade in Copenhagen (Denmark) (Caetano and Christensen, 1999). In 1991, Marja-Liisa Kaipio created a holistic program at the Käpylä Rehabilitation Centre (Kaipio et al., 2000; Sarajuuri and Koskinen, 2006). In November 1996, the Oliver Zangwill Center was inaugurated in Ely (England), thanks to the perseverance and tenacity of Barbara A. Wilson et al. (2000). In 2008, the ELEPAP-Rehabilitation for The Disabled Holistic Neuropsychological Rehabilitation Program was established in Athens in conjunction with the input of Dr. Ben-Yishay. In the first decade of the twenty-first century, a clinical research project is now being developed at the Virgen de las Nieves Hospital of Granada (Spain) (Caracuel Romero et al., 2005; Caracuel et al., 2012).

## Prigatano’s contributions to holistic approaches to neuropsychological rehabilitation

In the 1970s and 1980s, a multitude of cognitive rehabilitation programs proliferated to facilitate recovery after brain injury. However, only a few programs embraced the holistic approach. Greatly influenced by Diller and Ben-Yishay’s ideas and methods, Prigatano began, in early 1980, a holistic neuropsychological rehabilitation program at the Presbyterian Hospital in Oklahoma City (Oklahoma) (Prigatano et al., 1986) as noted above. In 1984, he published the first outcome paper on the effectiveness of a holistic neuropsychological rehabilitation program. Eighteen traumatic brain-injured patients enrolled in the program were compared to 17 untreated TBI patients. The groups were compared on cognitive tests, personality measures, emotional status, and employment measures. Treated patients had a higher incidence of employment, and their emotional status was considerably better than that of the untreated group. Ten years later, Prigatano and his colleagues provided empirical evidence that the therapeutic alliance with brain-injured patients and their families related to patients’ productivity status post rehabilitation. These findings provided evidence that holistic milieu-oriented neuropsychological rehabilitation approach for post-acute brain dysfunctional patients improved their psychosocial outcomes (i.e., increased the likelihood of employment and reduced level of emotional distress) (Prigatano, 1999).

Prigatano emphasized that it is artificial to separate cognitive and emotional disturbances in these patients, since how we feel affects how we behave and how we think. He argued that patients’ emotional and motivational reactions to their impairments greatly contribute to their degree of disability. He, like Goldstein and Ben-Yishay before him, stressed the importance of establishing a therapeutic milieu as a key component of the successful brain injury rehabilitation: “Group work alone does not define a holistic approach; rather, it is placing an individual in a therapeutic environment that influences psychosocial outcomes” (Prigatano, 1997, p. 497).

In 1999 Prigatano published *Principles of Neuropsychological Rehabilitation*, one of the seminal books in contemporary neuropsychological rehabilitation (Prigatano, 1999). Based on his experience at the Presbyterian Hospital and the BNI, Prigatano postulated thirteen principles that act as a guide for treatment of people with neuropsychological disorders (see Table 1). These principles are aimed at enabling individuals to regain a productive lifestyle and restore meaning to their lives. Prigatano highlighted the importance of the patient’s premorbid cognitive and personality functioning as determinants of neuropsychological rehabilitation process and outcomes. Emphasis was also placed on understanding the patient’s phenomenological experience in relation to their brain injury. Prigatano’s contributions facilitated our understanding of important ingredients in neuropsychological rehabilitation in adult brain-injured patients.

**TABLE 1:** Prigatano’s principles of neuropsychological rehabilitation.

Principle 1	The clinician must begin with patient’s subjective or phenomenological experience to reduce their frustrations and confusion to engage them in the rehabilitation process.
Principle 2	The patient’s symptom picture is a mixture of premorbid cognitive and personality characteristics as well as neuropsychological changes directly associated with brain pathology.
Principle 3	Neuropsychological rehabilitation focuses on both the remediation of higher cerebral disturbances and their management in interpersonal situations.
Principle 4	Neuropsychological rehabilitation helps patients observe their behavior and thereby teaches them about the direct and indirect effects of brain injury. This may help patients avoid destructive choices and better manage their catastrophic reactions.
Principle 5	Failure to study the intimate interaction of cognition and personality leads to an inadequate understanding of many issues in cognitive (neuro) sciences and neuropsychological rehabilitation.
Principle 6	Little is known about how to retrain a brain dysfunctional patient cognitively, because the nature of higher cerebral functions is not fully understood. General guidelines for cognitive remediation, however, can be specified.
Principle 7	Psychotherapeutic interventions are often an important part of neuropsychological rehabilitation because they help patients (and families) deal with their personal losses. The process, however, is highly individualized.
Principle 8	Working with brain dysfunctional patients produces affective reactions in both the patient’s family and the rehabilitation staff. Appropriate management of these reactions facilitates the rehabilitative and adaptive process.
Principle 9	Each neuropsychological rehabilitation program is a dynamic entity. It is either in a state of development or decline. Ongoing scientific investigation helps the rehabilitation team learn from their successes and failures and is needed to maintain a dynamic, creative rehabilitation effort.
Principle 10	Failure to identify which patients can and cannot be helped by different (neuropsychological) rehabilitation approaches creates a lack of credibility for the field.
Principle 11	Disturbances in self-awareness after brain injury are often poorly understood and mismanaged.
Principle 12	Competent patient management and planning innovative rehabilitation programs depend on understanding mechanisms of recovery and deterioration of direct and indirect symptoms after brain injury.
Principle 13	The rehabilitation of patients with higher cerebral deficits requires both scientific and phenomenological approaches. Both are necessary to maximize recovery and adaptation to the effects of brain injury.

With his emphasis on the need to clinically address the emotional and motivational disorders experienced by brain-injured patients, Prigatano highlighted the importance of psychotherapy in neuropsychological rehabilitation. When this idea was introduced in the 1980’s, many professionals thought that psychotherapy was contraindicated in brain-injured patients. Prigatano proposed that the “loss of normality” after brain injury can be understood as a loss of the possibility to fit into societal standards, cultural notions, or archetypes -in the Jungian sense- regarding what is considered desirable and valuable in human beings (i.e., beauty, intelligence, success) (Salas, 2021). Therefore, he posited that psychological interventions for brain injured survivors can incorporate Jungian insights in their care. It helps brain-injured patients explore new cultural symbols to find a place in the world and rebuild meaning in their life (i.e., work, love, and play) (Prigatano, 1989, 2012). Prigatano argued that psychological tools should be adapted to facilitate the reconstruction of the self after injury. Psychotherapy should teach the patient to learn to behave in his or her best self-interest. This does not mean selfish self-interest. It means learning to meet one’s own needs fairly and consistently.

Prigatano explicitly stated that psychotherapy could be of value to brain-injury survivors in several areas (Prigatano et al., 1986). It should attempt to:

• Provide a model that helps the patient understand what has happened to him or her.
• Help the patient deal with the meaning of the brain injury in his or her life.
• Help the patient achieve a sense of self-acceptance.
• Help the patient make realistic commitments to work and interpersonal relations.
• Teach the patient how to behave in different social situations.
• Provide specific behavioral strategies to compensate for neuropsychological deficits.
• Foster a sense of realistic hope.

Prigatano’s work with children with brain injuries is less well known. At the Brain Injury Rehabilitation Trust Biannual Conference, held in September 2011, Prigatano presented the paper *Challenges and opportunities facing holistic approaches to neuropsychological rehabilitation* (Prigatano, 2013). He points out that there are almost no post-acute holistic neuropsychological rehabilitation programs for brain-injured children. Children usually return home and/or to school with limited understanding regarding the management of their behavioral problems and the repercussion of their cognitive deficits. This situation not only has a negative impact on children but also on parents and teachers. Prigatano proposed that the effectiveness of the pediatric neuropsychological rehabilitation programs should be evaluated in four domains: (1) academic performance, (2) peer relationships, (3) parents’ level of anxiety, and (4) teachers’ level of anxiety. In brain-injured adults, the central problem is coping with lost normality and helping them to be productive and maintain interpersonal relationships, while fostering their own individuality. In children, the goal of post-acute neuropsychological rehabilitation is to facilitate normal developmental trajectories.

When commenting on the future of neuropsychological rehabilitation, Prigatano et al. (2021) proposed that neuropsychological rehabilitation is entering a new era. Understanding the mechanisms of change associated with improvement in higher integrative brain functions requires a close collaboration with scientists involved in understanding neuroimaging findings and markers of positive and negative neuroplasticity changes. In this regard, studying the complex phenomena of diaschisis, at both the cortical and subcortical levels, may well guide neuropsychological rehabilitation in the future just as Luria et al. (1969) originally suggested.

## Prigatano’s contributions to clinical neuropsychology

Prigatano is known principally for his contributions to holistic approaches to neuropsychological rehabilitation. However, his professional work has also influenced clinical neuropsychology.

While working at Presbyterian Hospital in Oklahoma City, Prigatano noticed that many of the patients showed an apparent lack of self-awareness of their cognitive and behavioral impairments and minimized the impact of their brain disorder on daily functioning (Prigatano, 1999). In October 1988, with Daniel L. Schacter he organized a scientific conference on the phenomenon of altered deficit awareness after brain injury. The papers of the conference were published in 1991 under the title *Awareness of Deficit after Brain Injury: Clinical and Theoretical Issues* (Prigatano and Schacter, 1991). This book brought the study of impaired awareness to the attention of many clinical neuropsychologists, who not had previously considered it to be an important area of clinical investigation.

Twenty years later, in October 2008, Prigatano organized a second meeting to address anosognosia and related syndromes. Two years later, he published *The Study of Anosognosia* (Prigatano, 2010). In the preface of that book, Prigatano stated that the study of anosognosia is important for several reasons:

– Patients with brain disorders may have reduced self-awareness of residual neurological or neuropsychological impairments that negatively impact their clinical care.
– Understanding the biological and neuropsychological mechanisms responsible for anosognosia in its various forms may reveal important insights into brain organization and how human consciousness is possible.
– The comparative study of anosognosia in patients with brain disorders in comparison to patients with psychiatric disorders may provide rich insights into the “body-mind” problem.
– Understanding the basis of how anosognosia changes with time (i.e., “recovers” or “worsens”) may provide important insights into mechanisms of “recovery” and “deterioration” after various brain disorders. This has clear implications for treatment and patient management.

In bringing attention to the fact that many people with different brain disorders overestimate their abilities, presumably because of their altered awareness of deficits, he also developed, in collaboration with his colleagues in Oklahoma City, the Patient Competence Rating Scale (PCRS), which was designed as a scale to determine awareness of deficits after brain injury (Prigatano et al., 1986). Following a discrepancy-based approach, the PCRS is administered to the individual with a brain injury and an informant (e.g., family member or rehabilitation professional). Both are asked to judge the individual’s ability to do a variety of everyday tasks. The resulting total discrepancy score indicates the overall level of self-awareness. Currently the PCRS is one of the most widely utilized questionnaire-based measures of self-awareness (Kolakowsky-Hayner, 2010).

Prigatano (1999) observed that traumatic brain injured patients who finger tapped slowly in Halstead Finger Tapping Test tended to overestimate their behavioral competence in comparison with relatives’ reports. Years later, Prigatano (1999) demonstrated that speed of finger tapping may be a useful behavioral marker for predicting goal attainment during early stages of neurorehabilitation after a unilateral stroke. These findings led Prigatano to consider that this apparently “simple” task of speed of finger movement would be sensitive to the severity of disturbances of self-awareness and rehabilitation outcome (Prigatano, 1999).

Among the best known, and most used, cognitive screening tests are the Mini-Mental State Examination (MMSE) (Folstein et al., 1975) and the Montreal Cognitive Assessment (MoCA) (Nasreddine et al., 2005). The usefulness of these two cognitive screening tests in neurorehabilitation, however, has been questioned. In the 1990s Prigatano created a specific a cognitive screening test to assess patients with brain injuries called The Barrow Neurological Institute Screen for Higher Cerebral Functions (BNIS) (Prigatano et al., 2022). The BNIS includes an assessment of affect and awareness, along with measures of various cognitive domains. This broader scope account for the BNIS’s more accurate predictions of rehabilitation outcomes. It also demonstrated that disturbances in affect, as measured by this screening test, were sensitive to various brain disorders.

His influence in clinical neuropsychology, therefore, was to expand the scope of a traditional neuropsychological assessment to include assessments of awareness and affect. He also demonstrated that impairments in self-awareness exist in various patient groups even when frank anosognosia is not present. Finally, his research on finger tapping emphasizes the need to have “simple,” direct measures of behavior that can monitor the recovery and deterioration of brain function over time. This perspective expanded the role of neuropsychological assessment from diagnosis to prognosis.

## Autobiographical summary (Prepared by GPP)

I was born on September 13, 1944. My father was the product of the Great Depression and left school in the 8th grade to work to save the family’s home. My mother was born in Sicily and moved to the United States at age 4. One of her brothers became a physician while the other became a chemist trained at Princeton University. This second brother (i.e., Uncle Joe) spent a lot of time with me during my childhood and served as an important role model who emphasized the importance of education, working hard, and speaking one’s mind. My father and his father emphasized the very same values as I was growing up.

Being a product of an Italian-American family, I was raised Catholic and attended Catholic grammar school and high school. At around age 8, I read about the life of “Father Damien.”^1^ It had a deep personal significance for me. It was a true “hero’s story” that moved me consciously and unconsciously into a clinical profession.

In college, I was educated by Jesuit priests who emphasized the importance of the individual to think for themselves when making important life decisions. Those teachers also emphasized, in my philosophy courses at Loyola University in Los Angeles (1962–1966), that no theory is “right” or “wrong.” The usefulness of a theory depends on its ability to explain or predict a given phenomenon. This instilled a questioning attitude toward any “dogma” which told clinical neuropsychologists how to practice their profession.

### Early influences in the study of psychology

During my undergraduate education, both philosophy and psychology were emphasized. I was therefore very much influenced by the debate been Carl Rogers and Burrhus Frederic Skinner at that time. It was true that behavior is a function of its consequences (as Skinner scientifically demonstrated). However it was also true that entering the patient’s subjective experiences, in order to help them clarify how they are experiencing themselves and the world around them, could be very important in helping them find meaning in their lives and reduce their psychological suffering in its many forms (i.e., anxiety reactions, depression, angry outbursts, impaired interpersonal relationships etc.) as Carl Rogers quietly but persuasively stated.

During pre-doctoral academic training as a clinical psychologist, my teachers emphasized the role of learning-based techniques for reducing behavioral problems. I was taught such procedures as systematic desensitization to reduce certain types of anxiety or avoidance behaviors in college students seeking services from the Department of Psychology’s Psychological Clinic. The theories of Freud, Jung and Adler were glossed over in my academic doctoral training program and considered having no scientific and little clinical value.

Later, during my pre-doctoral clinical internship at the University of Oklahoma Health Science Center, I was confronted with the real problems of psychiatric patients (i.e., alcohol abuse, psychosis, severe depression and anxiety, severe personality disorders, etc.). Listening to these patients’ life stories and current struggles, it became clear that the teachings of Rogers and Skinner were only partially helpful. The teachings of Freud, Jung, and Adler suddenly seemed to be very relevant to these psychiatric problems.

I was fortunate to obtain excellent supervision from experienced psychiatrists particularly influenced by Freudian and Neo-Freudian theories during this time. The role of unconscious influences on behavior now became clearer to me; working with these influences seemed to produce meaningful behavioral changes and a reduction of personal (psychological) suffering in some of the patients I helped treat.

During my pre-doctoral clinical psychology internship, I was also introduced to the field of neuropsychology by Oscar Parsons, who emphasized the importance of writing a clear neuropsychological report and publishing one’s findings in a concise manner. This influence persists in my professional life as a diagnostician, a psychotherapist, and a researcher.

### Post-doctoral influences

Many individuals had a substantial impacted my clinical and research work, as acknowledged in previous publications (Prigatano, 1999, 2019). In the area of neuropsychology assessment, the work of Ward Halstead and Ralph Reitan was especially influential, particularly when it came to choosing neuropsychological tests to administer to patients when attempting to answer the old question of whether there were reliable and valid behavioral indicators of underlying brain pathology. Hearing Dr. Reitan tell clinical neuropsychologists at professional meetings that they were not very good at assessing individual differences in normal neuropsychological functioning left a lasting impression on me. I was further influenced by Gertrude Baker’s use of projective techniques when evaluating persons suspected of an “underlying organic brain syndrome” (Baker, 1956). Her work provided unique information as to what a brain dysfunctional person experiences when confronting vague or unclear visual stimuli.

I also found the writings of Karl Pribram especially interesting since he discussed Freudian concepts within the context of the neurosciences (Pribram, 1962; Pribram and Gill, 1976). After presenting a paper at a conference in Oxford, England, I met Karl Pribram. I expressed my interest in understanding how memory and emotion are organized in the brain and how they are affected by various brain disorders. Dr. Pribram invited me on the spot to come to Stanford and study with him. It was not a difficult decision but a risky one. Having just been promoted to a tenured position as an Associate Professor, I would have to leave that security to purse what was most meaningful to me. With the support of Dr. R. Barton Carl, neurosurgeon, that decision was made, which was a life changing event for me.

In my initial efforts at holistic neuropsychological rehabilitation with post-acute young adults with severe traumatic brain injury, I became impressed that many of these patients seemed to not be aware of or were denying the presence and severity of various neuropsychological impairments. Reviewing the existing literature at the time, I came across Weinstein and Kahn’s (1955) book *Denial of Illness: Symbolic and Physiological Aspects.* It was the only book that seem to address this important issue. I asked Edwin Weinstein to participate in an American Psychological Association symposium I had organized on neuropsychological rehabilitation. Weinstein accepted the invitation, and we began several dialogues about denial phenomenon. Weinstein’s insights helped clarify an important clinical and theoretical question. Was it possible for a brain dysfunctional person to have altered self-awareness of a neuropsychological disturbance due to the direct effect of a brain disorder (i.e., anosognosia or impaired self-awareness) and at the same time minimize the presence or extent of a neuropsychological impairment as a method of coping with anxiety over the presence of that impairment (i.e., defense denial)? That question lead to a long series of studies done with colleagues to help arrive at a reasonable answer. The answer was “yes.”

Undoubtedly, however, the biggest influence on my work in neuropsychological rehabilitation came from my professional interactions with Leonard Diller and Yehuda Ben-Yishay. The development of a holistic approach that including both cognitive rehabilitation and psychotherapy now became a passion for me. Dr. Ben-Yishay played a very special role in helping me organize a day treatment program and provided me with a methodology for approaching the cognitive rehabilitation of patients with chronic brain injuries who had not benefited from traditional forms of rehabilitation.

Being impressed with the importance of impairments of self-awareness in the rehabilitation process, I worked with patients to determine if their emotional state could be improved. However, it was unclear to me as to the best way of approaching this domain of the patients’ lives. Early in our rehabilitation work, a young man presented me with a spontaneous drawing, which reflected his phenomenological experience of his brain injury on his life and how he was attempting to come to grips with the consequences of his brain injury in the most productive manner. A picture of the drawing is found in Prigatano et al., 1986, p. 85, Figure 5.4. The picture captured many important themes, but most importantly the artistic work of the patient presented new insights as to what many brain dysfunctional patients personally struggle with following their injuries. It provided insights as to how to best help them with their emotional and motivation struggles. The role of individual and group psychotherapy was now firmly established in the holistic programs of neuropsychological rehabilitation I developed (both in Oklahoma City and Phoenix, Arizona).

Many individuals influenced my thinking about psychotherapy. They are listed by name in the Preface of my most recent book (Prigatano, 2019). By and large, however, it was the writings of Carl Gustav Jung and his trainees that have had the greatest impact. After reading Bach’s (1969) book *The Spontaneous Paintings of Severely ill Patients: A Contribution to Psychosomatic Medicine*, I sought out a personal consultation with Dr. Bach, a Jungian analyst living in London. The discussion left an indelible mark on how I thought about the unconscious and how it influences various art expressions that can inform the psychotherapist about what the patient is experiencing. These art forms often point to the next step in their psychological development as clearly described in Kalff’s (1980) book *Sandplay*.

These professional dialogues lead to several papers on the importance of symbolism in the neuropsychological rehabilitation process. Helping patients relate to their symbols of work, love and play guided psychotherapeutic efforts when supporting their return to a productive lifestyle and re-establishing meaning in their lives. Symbols involving the hero’s journal now became an important prototype of the psychotherapeutic process for many individuals (Prigatano, 1999). It continues to influence my present-day psychotherapeutic work with patients with various brain disorders as well as functional movement disorders.

### Summary comments

The intent of this paper was to first summarize the contributions of George P. Prigatano during his 50th year of practice from the perspective of a part-time amateur historian and to then to combine that perspective with Prigatano’s own views of the primary influences on his work. Normally, the person’s professional career is reviewed by others, and the person’s own view of what influenced their work is not present. We believe both sources of information can be useful in historical accounts. It is our hope that this paper has achieved that goal.

At the conclusion of this paper, we include an Addendum that summarizes George P. Prigatano’s professional career to date.

## Addendum: Brief review of George P. Prigatano’s 50 year career

George P. Prigatano obtained his Ph.D. in Clinical Psychology on August 31, 1972 from Bowling Green University in Ohio. He attended Bowling Green University because it offered a joint Ph.D. degree in both Industrial and Clinical Psychology, but soon his interests turned to Clinical Psychology. During his pre-doctoral clinical psychology internship at the University of Oklahoma Health Sciences Center, he was introduced to a new field of study called neuropsychology. After completing his Clinical Psychology Internship, he became an Assistant Professor of Psychiatry and Behavioral Sciences and was the Director of the Neuropsychology Testing Laboratory. In 1978, he was promoted to Associate Professor (Tenured), but left his academic position to study neuropsychology with Karl Pribram, M.D. at Stanford University. Following that time, he developed a Neuropsychology Rehabilitation Program at Presbyterian Hospital in Oklahoma City (1979–1985). He was recruited to the BNI in Phoenix Arizona in 1985 to begin the Department of Clinical Neuropsychology and to establish a Holistically Oriented Neuropsychological Rehabilitation Program like the one he developed at Presbyterian Hospital in Oklahoma City. That program was influenced and supported of two key figures -Yehuda Ben-Yishay, Ph.D. and R. Barton Carl, M.D.-. He was the Founder and first Chairman of the Department of Clinical Neuropsychology at the BNI from 1985 to 2014. From 2014 to this date, he has been the Emeritus Chairman and has remained clinically and academically active. He served as the President of the National Academy of Neuropsychology in 1998. In conjunction with Neil Pliskin edited the first book on the importance of cost outcome research for the future of clinical neuropsychology (Prigatano and Pliskin, 2003).

Over the course of his career, Dr. Prigatano has lectured nationally and internationally on the various topics listed above. His most recent workshop was held at the National Academy of Neuropsychology meeting in December of 2021. He spoke on the important role of providing psychological care for persons with various brain disorders. The lecture was devoted to the memory of Leonard Diller and Yehuda Ben-Yishay.

## Author contributions

All authors listed have made a substantial, direct, and intellectual contribution to the work, and approved it for publication.

## Footnotes

1 Father Damien (1840–1889) was a Belgian priest who worked as a missionary at the Molokai leprosy settlement (Hawaii’s fifth largest island). In 1884 he contracted leprosy. Even though the illness was slowing his body down, Damien engaged in a lot of activities during his last years.
